# Hybrid biomimetic assembly enzymes based on ZIF-8 as “intracellular scavenger” mitigating neuronal damage caused by oxidative stress

**DOI:** 10.3389/fbioe.2022.991949

**Published:** 2022-08-31

**Authors:** Qing Li, Ruixia Feng, Zhaohui Chang, Xiaojun Liu, Hao Tang, Qian Bai

**Affiliations:** ^1^ Department of Molecular Pathology, Application Center for Precision Medicine, The Second Affiliated Hospital of Zhengzhou University, Zhengzhou, China; ^2^ Department of Critical Care Medicine, Second Affiliated Hospital of Zhengzhou University, Zhengzhou, China; ^3^ National Health Commission Key Laboratory of Cardiovascular Regenerative Medicine, Heart Center of Henan Provincial People’s Hospital, Central China Fuwai Hospital of Zhengzhou University, Fuwai Central China Cardiovascular Hospital and Central China Branch of National Center for Cardiovascular Diseases, Zhengzhou, China

**Keywords:** biomimetic mineralization, SOD@ZIF-8, nerve damage, ROS scavenging, neurological disorders

## Abstract

Superoxide dismutase (SOD) was immobilized in zeolite imidazolate framework-8 (ZIF-8) through biomimetic mineralization method, namely SOD@ZIF-8, which was then used in the treatment of nerve damage by eliminating reactive oxygen species (ROS). A series of chemical characterization and enzymatic activity researches revealed that SOD was successfully embedded into ZIF-8 without apparent influence on the antioxidant activity of SOD. Cell level experiments showed that SOD@ZIF-8 could be effectively endocytosed by cells. The activity of SOD@ZIF-8 in scavenging ROS played a critical role in protecting SHSY-5Y cells from MPP^+^-induced cell model and relieving cell apoptosis, indicating that SOD@ZIF-8 could effectively rescue ROS-mediated neurological disorders though removing excessive ROS produced *in vitro*.

## Introduction

Reactive oxygen species (ROS), which is frequently mentioned in the field of biology and medicine, refers to oxygen-containing substances with high reactivity, specific including superoxide anion (O_2_^−^), hydrogen peroxide (H_2_O_2_), hydroxyl radical (OH˙), singlet oxygen (^1^O_2_), peroxide free radical (LOO˙), hydrogen peroxide lipid (LOOH), peroxynitro group (ONOO^−^), hypochlorous acid (HOCl), ozone (O_3_) and so on. ROS generated in the process of oxygen metabolism are essential for cell signaling and immune response (Finkel, 2011; Sena and Chandel, 2012). However, the excessive expression of ROS is deleterious especially in the brain, since it induces oxidative damage of protein, lipid and DNA. As such, ROS are highly responsible for the development of many nerve damage such as cerebral palsy, Alzheimer’s diseases, schizophrenia and Parkinson’s diseases (PD) (Dietrich-Muszalska and Kontek, 2010; Popa-Wagner et al., 2013; Yan et al., 2013). Superoxide dismutase (SOD), as a crucial member of the antioxidant system, can efficiently catalyze the decomposition of superoxide radical to hydrogen peroxide and oxygen (Hall et al., 2010; Hu and Tirelli, 2012; Van Raamsdonk and Hekimi, 2012). Therefore, it has demonstrated therapeutic potential in the treatment of ROS-mediated diseases including inflammation, diabetes, radiation injuries, and central nervous system (CNS) disorders (Seshadri et al., 2010; Wang et al., 2010). However, due to the relatively instability character and short intracellular half-lives of proteins, direct use of SOD has been hampered by poor pharmacokinetics, rapid renal clearance, degradation by proteases in the serum and low cellular membranes penetrating ability (Tong et al., 2012; Chen et al., 2013; Liu et al., 2021a; Li et al., 2022a). Thus, to enhance SOD stability and bioavailability with desirable catalytic activity, immobilization method has been investigated by using different solid supports such as organic polymers, magnetic nanoparticles and mesoporous silica (Wu and Yang, 2017; Li et al., 2021). Among these materials, metal-organic frameworks (MOFs), consisting by metal ions nodes and organic ligands, have been increasingly proven to be a robust solid support in the enzyme immobilization due to its large surface area, tunable porosity and functionality, favorable excellent thermal/mechanical stability (Orellana-Tavra et al., 2015; Lian et al., 2016; Lian et al., 2017; Abdelhamid, 2021; Hoseinpour and Shariatinia, 2021; Li et al., 2022b). Generally, MOF-based immobilization approaches are mainly included by physical adsorption or entrapment, which have demonstrated unique advantages such as high loading efficiency of enzymes and excellent stability against high temperature and organic solvents (Ding et al., 2021). Nevertheless, these strategies are not able to achieve the size control of enzyme-MOF composites and maintain the enzymatic activity due to MOF-induced destabilization of enzymes’ conformation. Recently, biomimetic mineralization approach for MOF synthesis has been adopted to encapsulate the enzyme molecules within protective exteriors through self-assembly process (Liang et al., 2015; Chen and Ma, 2016; Liang et al., 2016; Mehta et al., 2016; Abdelhamid, 2020; Fan et al., 2021). These enzyme-MOF composites are able to solve the unfavorable solvent effect of enzymatic structure and limited loading of large enzyme into MOF pores. Unlike other encapsulation approaches such as coprecipitation, enzyme molecules can trigger nucleation of porous crystals by concentrating the MOF building blocks in the process of biomimetic mineralization (Xie et al., 2010; He et al., 2016; Jiang et al., 2017; Zhu et al., 2022). Notably, enzyme-MOF composites obtained from this method can enlarge the bioactive temperature range of enzyme and enhance the stability in a facile manner, demonstrating a great promise in the biomedical application (Montanari et al., 2012; Tabrez et al., 2012; Zhu et al., 2012; Li et al., 2020; Yang et al., 2020).

**SCHEME 1:**
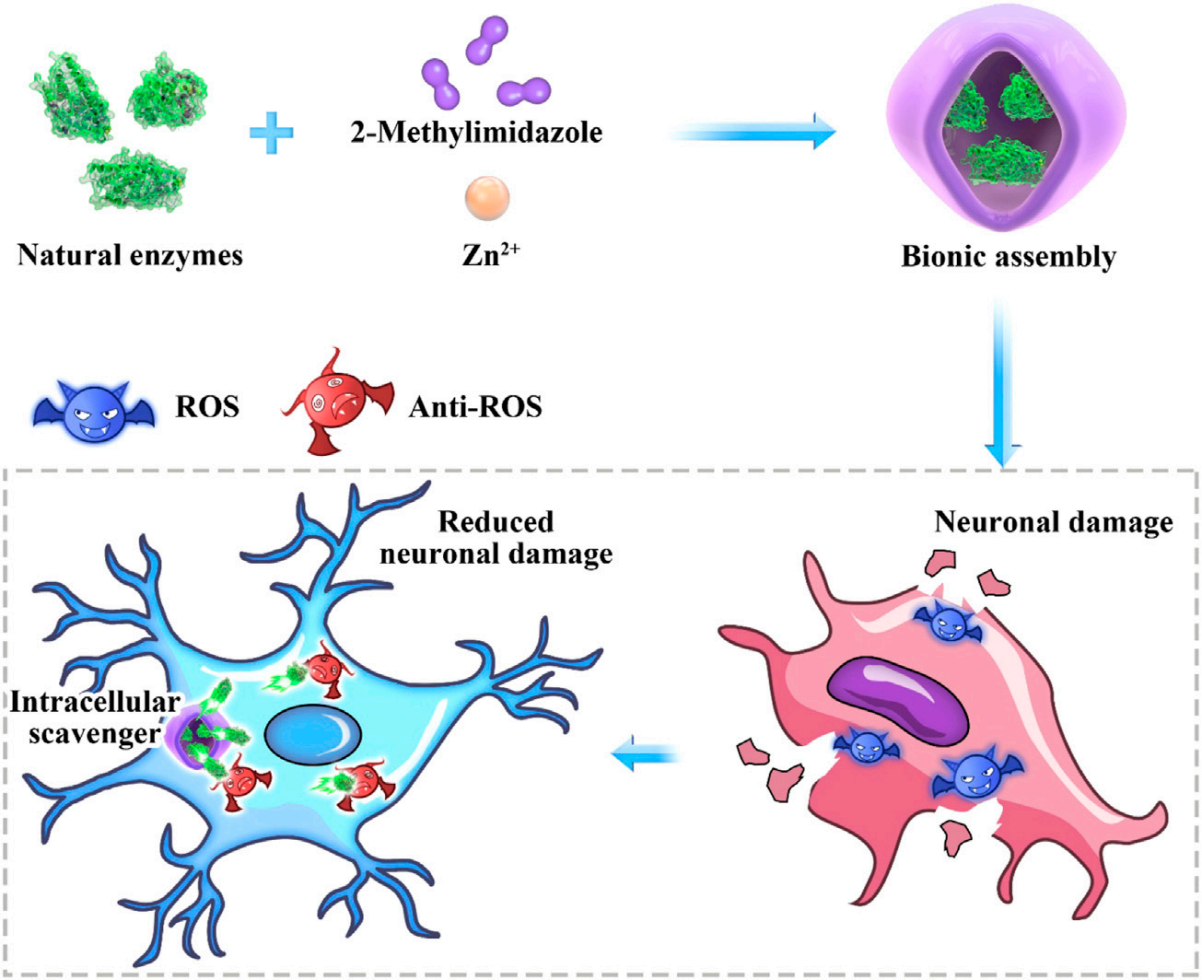
Antioxidant biomimetic assembly as “intracellular scavenger” for the treatment of neuronal damage.

Herein, SOD was embedded into zeolite imidazolate framework-8 (ZIF-8) through biomimetic mineralization method (denoted as SOD@ZIF-8). The SOD@ZIF-8 assembly was then employed as ROS scavenger in neuronal damage treatment. Significantly, a n neuronal damage cell model was established to evaluate the therapeutic effect of SOD@ZIF-8 (Scheme 1).

## Results and discussion

**FIGURE 1:**
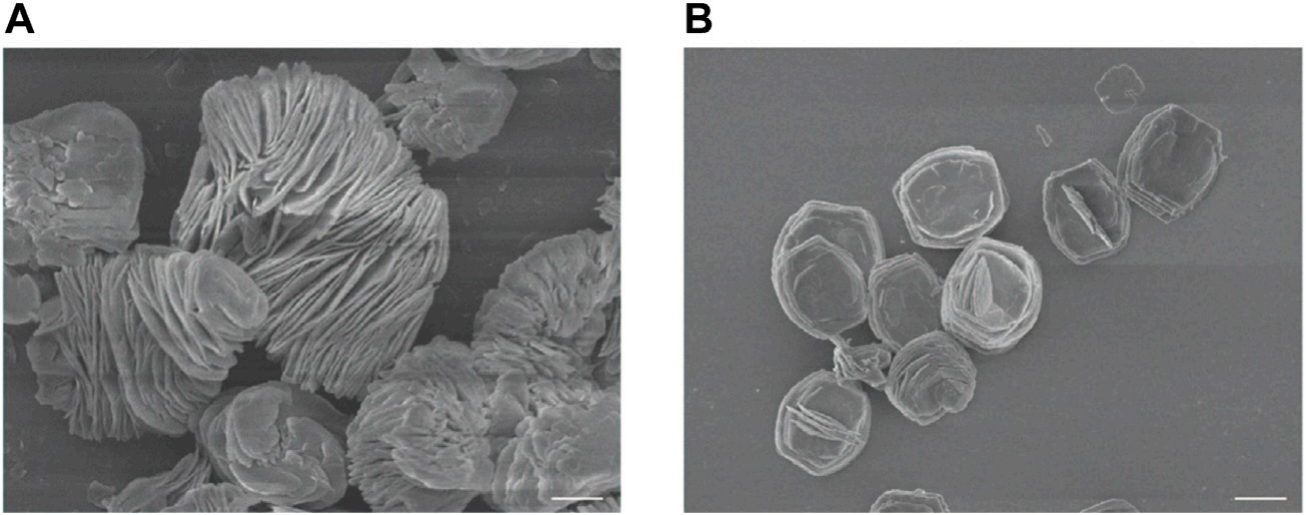
SEM images of ZIF-8 with the molar ratios of 2-dimethylimidazole and zinc acetate at 4:1 **(A)** and 24:1 **(B)**. The scale bars are 5 μm.

**FIGURE 2:**
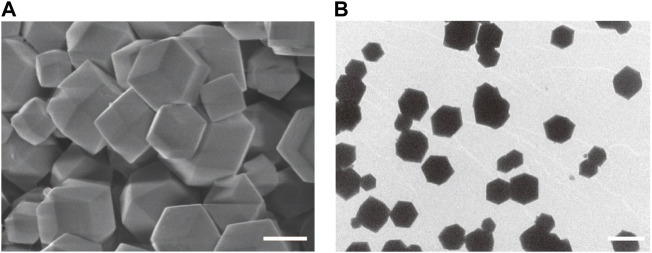
SEM images **(A)** and TEM images **(B)** of SOD@ZIF-8. The scale bars are 100 nm for A and 200 nm for **(B)**

**FIGURE 3:**
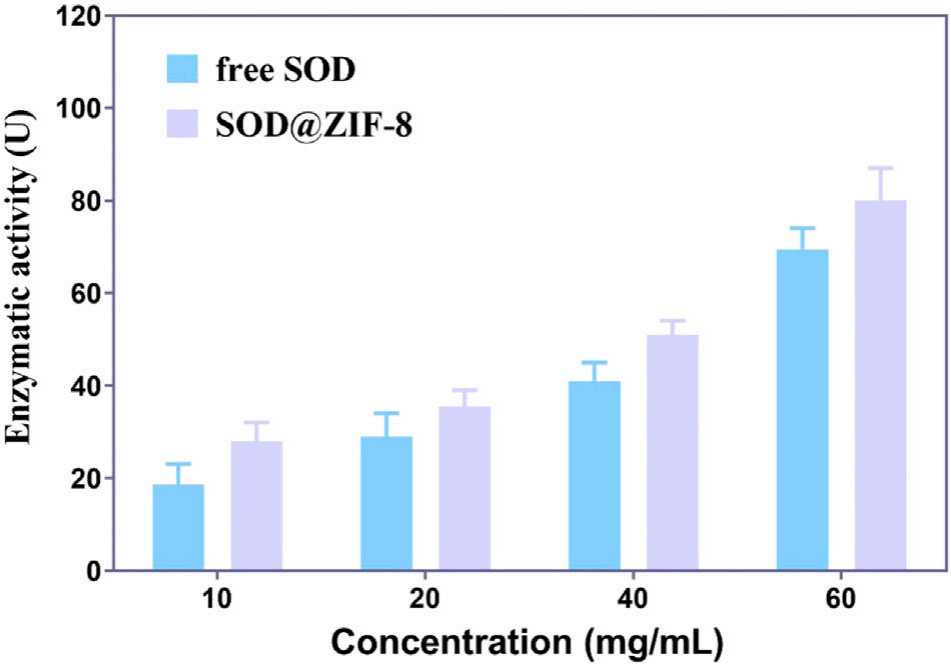
The enzymatic activities of free SOD and SOD@ZIF-8 under various concentrations.

Firstly, ZIF-8 with various molar ratios of 2-dimethylimidazole/Zn^2+^ (4:1, 24:1 and 48:1) were synthesized to investigate the suitable morphology and particle size for biomedical applications. As shown in Figure 1, micro-scaled SOD@ZIF-8 composites with size of more than 3 μm were obtained at the molar ratios of 4:1 and 24:1, respectively. Moreover, their morphology is too irregular to illustrate a single assembly enzyme complex. When the molar ratio was fixed at 48:1, regular dodecahedron structure and smaller size of SOD@ZIF-8 with 150–200 nm was achieved, which was preferable for the biomedical applications. Further, SOD was immobilized into ZIF-8 through biomimetic mineralization method. SEM and TEM images (Figure 2) suggested that the morphology and particle size did not change significantly compared with me-ZIF-8. Importantly, after ZIF-8 immobilization, the SOD activity was improved compared with free SOD (Figure 3), suggesting the biomimetic mineralization stategies could help enhance the enzymatic activities of SOD. This phenomenon might result from the fact that ZIF-8 benefit the dispersion of SOD, thus exposing more enzyme-activity centers and promoting enzymatic activity.

**FIGURE 4:**
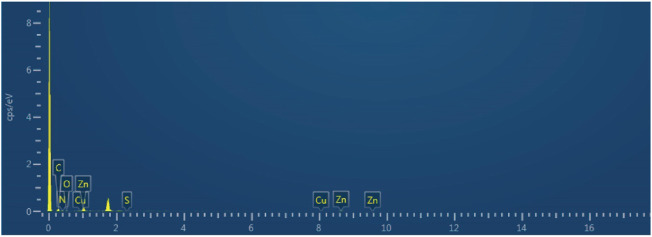
Elemental analysis of SOD@ZIF-8.

**FIGURE 5:**
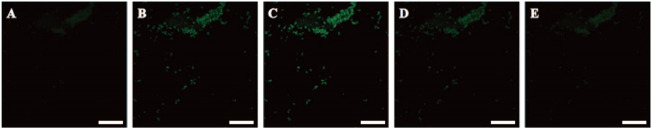
CLSM photographs of FITC-SOD@ZIF-8 with various depth at the z-axis of **(A)** 50 nm, **(B)** 100 nm, **(C)** 150 nm, **(D)** 200 nm, **(E)** 250 nm. 488 and 530 nm were chosen as the wavelengths for the detection of the fluorescence of FITC. The scale bars are 200 μm.

Meanwhile, chemical elements analysis of SOD@ZIF-8 showed the presence of Cu signal in the composites, presenting immobilization of enzyme harboring Cu element in ZIF-8 through biomimetic mineralization (Figure 4). To further validate the efficient assembly of SOD enzyme, SOD was labeled with fluorescein isothiocyanate (FITC) and then incorporated into ZIF-8 to construct FITC-labeled SOD@ZIF-8 through biomimetic mineralization. As shown in (Figure 5), green fluorescence of FITC-labeled SOD was clearly shown in the SOD@ZIF-8 assembling enzyme through laser confocal microscope. The fluorescence intensity of FITC showed a gradually increasing trend from the peak to the middle and then reduced to the bottom, indicating the successful incorporation of SOD into ZIF-8. Besides, The BCA result suggested that the loading efficiency of SOD in SOD@ZIF-8 was 23.43 wt%, which was a relatively high level among the reports of SOD immobilization.

**FIGURE 6:**
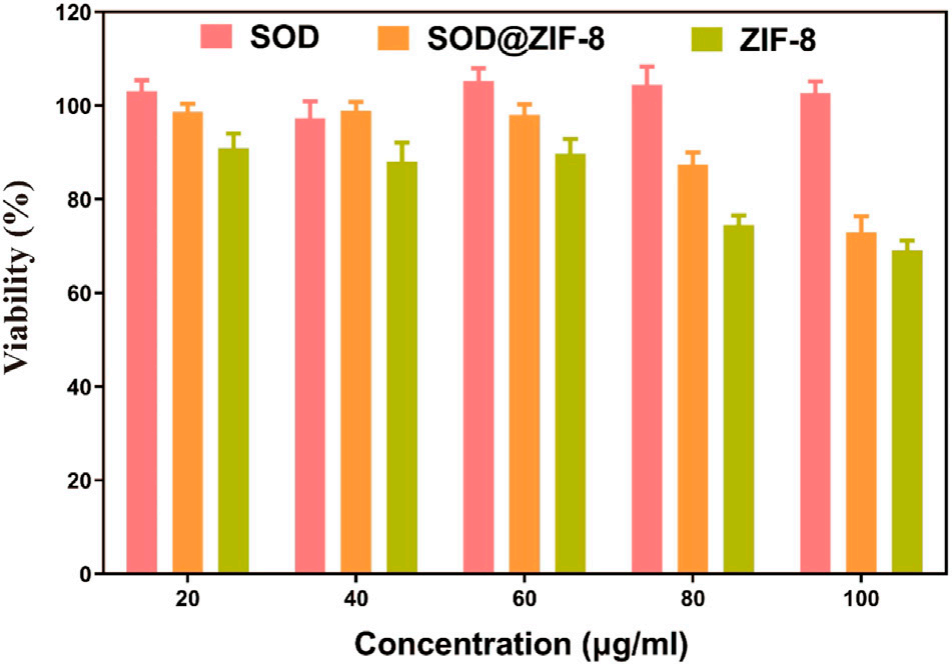
Toxicity of drugs with the same SOD content on SHSY-5Y cells. The concentrations of SOD@ZIF-8 varied from 20 to 100 μg/ml.

**FIGURE 7:**
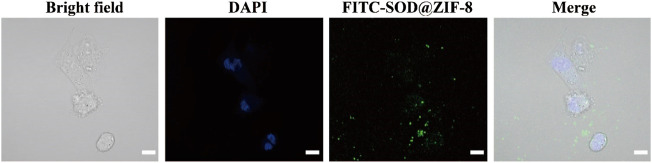
Cellular uptake ability of SOD@ZIF-8 detected by CLSM.

To investigate the applications of SOD@ZIF-8 composites *in vitro*, cell viability was examined to determine their potential cytotoxicity through MTT assay. As shown in Figure 6, compared with pure ZIF-8, the entrapment of SOD into ZIF-8 through biomimetic mineralization significantly reduced the cytotoxicity of ZIF-8 at various concentrations of 20–100 μg/ml. Negligible cytotoxicity was observed in SOD@ZIF-8 composites at the concentration of 60 μg/ml, indicating that SOD@ZIF-8 presented a satisfactory biocompatibility against cells. Subsequently, SOD was labeled with FITC and then employed to determine the cellular uptake of SOD@ZIF-8 by fluorescence microscope. As shown in Figure 7, strong green fluorescence was observed in FITC-SOD@ZIF-8 nanocomposites-treated cells, and It can be clearly found that the particles are located in the cell rather than on the cell surface, verifying that SOD@ZIF-8 were efficiently uptake by cells. The superior endocytosis of SOD@ZIF-8 composites by cells was mainly attributed to the nanosized structure, which was essential for intracellular release of SOD to cleave ROS *in vitro*.

**FIGURE 8:**
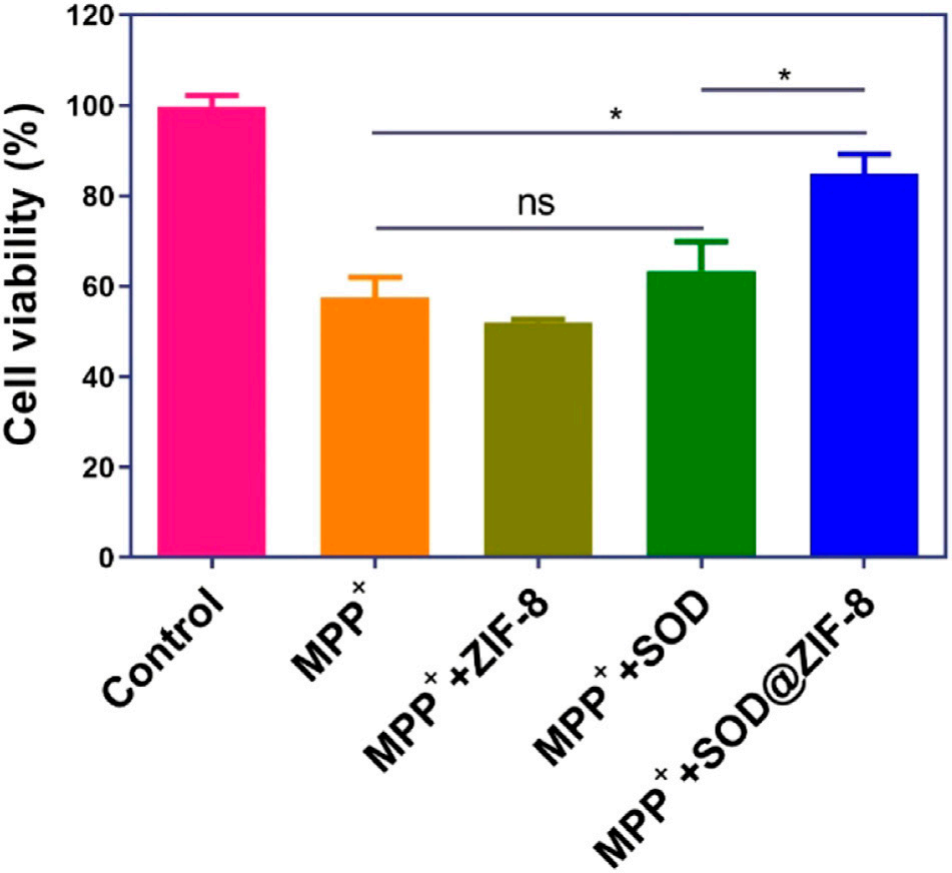
Viability of SHSY-5Y cells exposed to MPP^+^ after different treatments.

**FIGURE 9:**
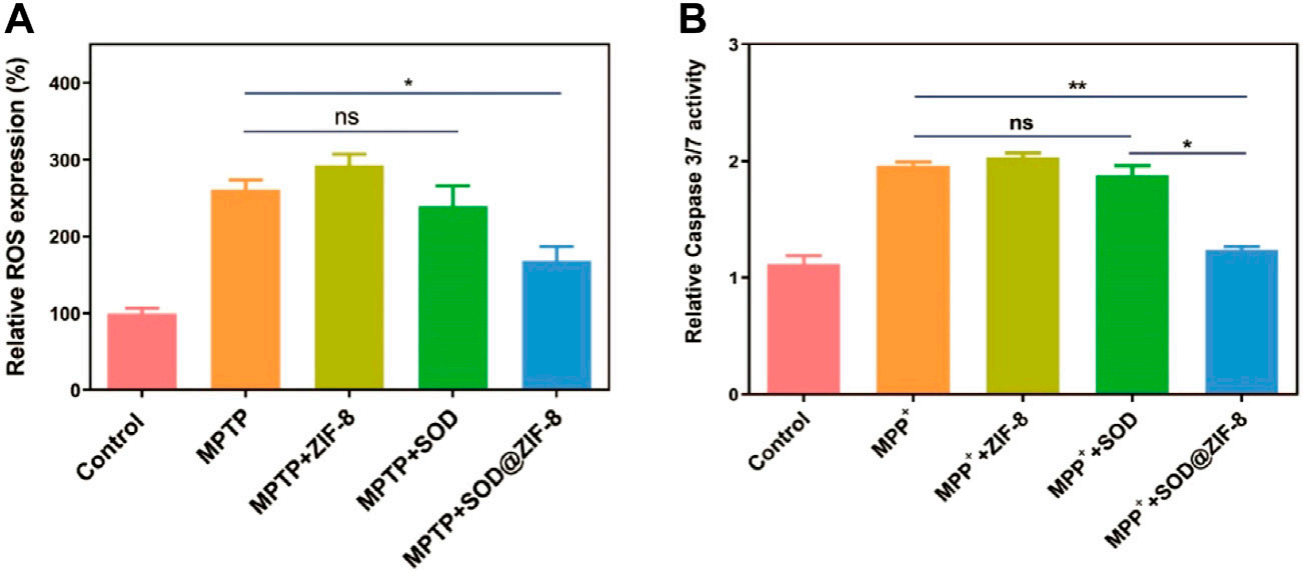
ROS scavenging activity of various nanomaterials in MPP^+^ treated SHSY-5Y cells detected by flow cytometry with DCFDA-H2 dye **(A)** and the detection of apoptosis by measuring the activity of effector caspase-3/7 **(B)**.

For these encouraging advantages, we then investigated the ROS scavenging ability of SOD@ZIF-8 composites. 1-Methyl-4-phenylpyridinium (MPP^+^) as a neurotoxin was able to cause cell death in human neuroblastoma SH-SY5Y cells due to the intracellular elevation of ROS and apoptosis induction, which typically was used to resemble nerve damage phenotype (Ding et al., 2021). As shown in Figure 8, obvious cell death was exhibited after SH-SY5Y cells were incubated with 2 mM MPP^+^. In contrast, cells pre-treated with SOD@ZIF-8 composites demonstrated an increasing viability after exposure to MPP^+^, confirming the protection afforded by SOD@ZIF-8 composites against neurotoxin. Subsequently, 2′-7′-dichlorodihydrofluorescein diacetate (DCFH-DA), which was used to directly measure the redox state of cells, were adopted to detect ROS generation of SH-SY5Y cells. As shown in Figure 9A, SH-SY5Y cells treated with MPP^+^ exhibited an enhanced expression of ROS, leading to the cell death. Meanwhile, a significant decrease of ROS expression was achieved after SOD@ZIF-8 composites treatment, which was in agreement with MTT results. To confirm the protective effect of SOD@ZIF-8 composites, the activity of caspase-3 was detected. As illustrated in Figure 9B, compared with untreated cells, MPP^+^ treatment triggered caspase-3 activation, leading to the apoptotic death of SH-SY5Y cells. However, a significant decrease of caspase-3 expression was achieved in SOD@ZIF-8 treatment, revealing that SOD@ZIF-8 composites inhibited apoptotic effect by downregulation of caspase-3.

**FIGURE 10:**
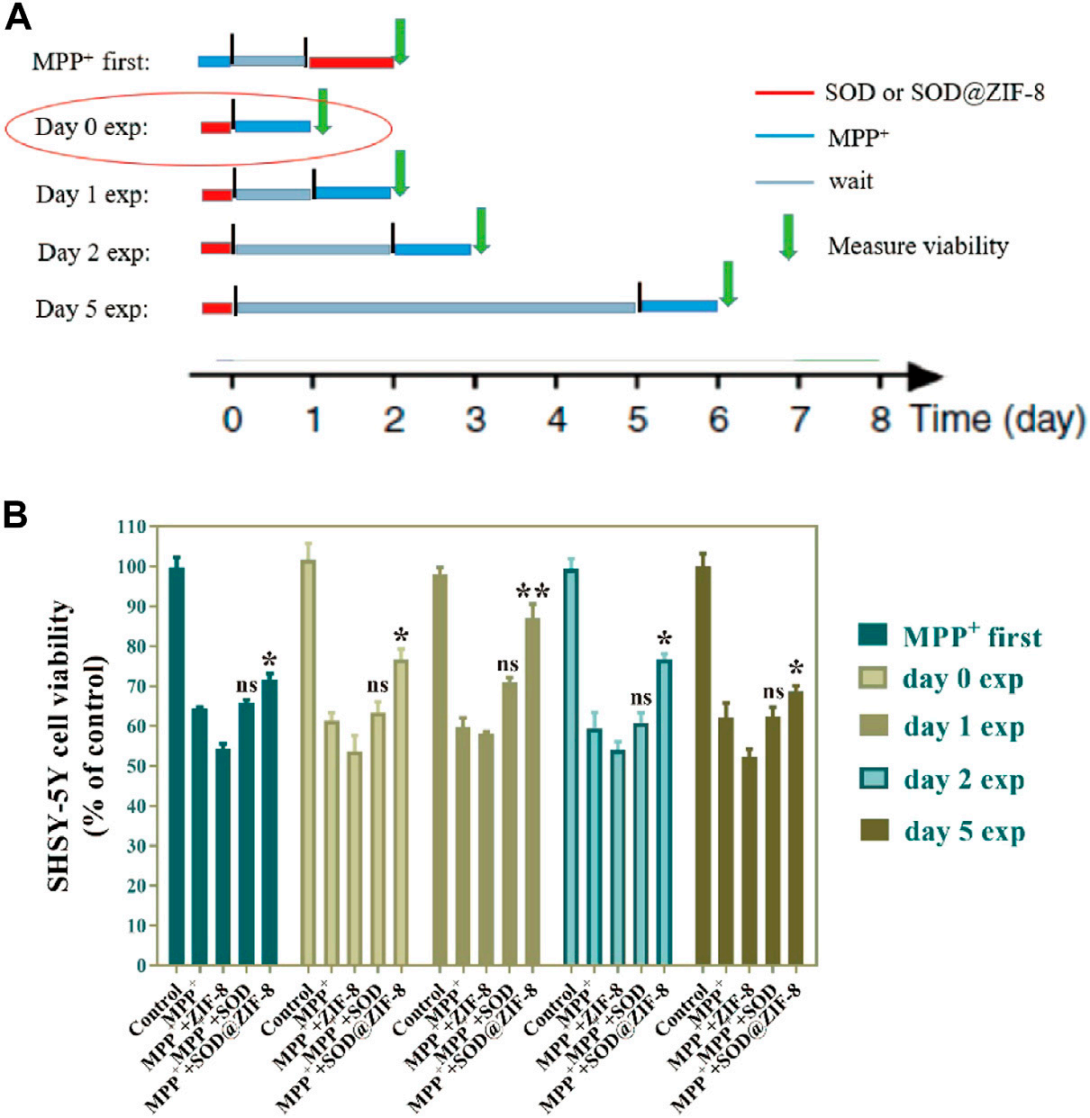
Long-term persistence effect of SOD@ZIF-8. Cells were pretreated with MPP^+^ (MPP^+^ first)or pretreated with SOD@ZIF-8 (Day 0 exp to Day 5 exp), washed and continued to culture for several days (gray time period in schematic diagram) and then exposed to MPP^+^ for 24 h. Cell viability was quantified by MTT assay.

To assess the long-term therapeutic effect of SOD@ZIF-8 composites, SH-SY5Y cells were incubated with SOD@ZIF-8 in advance, subsequently treated with MPP^+^ following several days. As shown in Figure 10, SOD@ZIF-8 composites achieved a better protective effect compared with free SOD during 1, 2 and 5 days. Meanwhile, the protective effect afforded by SOD@ZIF-8 composites was gradually decreased in a time-dependent manner, where still 70% cell viability was achieved after 5-days treatment. These results could confirm that biomimetically mineralized enzymes maintained a persistent protective effect for cells. Interestingly, simultaneous administration of SOD@ZIF-8 composites and MPP^+^ demonstrated a little lower protective effect, which was probably caused by the delayed release of SOD from SOD@ZIF-8 composites after cellular uptake. Similarly, less protective effect was achieved when cells were pre-incubated with MPP^+^ and then treated with SOD@ZIF-8, indicating that SOD@ZIF-8 composites was attributed to prevention of MPP^+^-induced cell damage. These results demonstrated that biomimetically mineralized SOD@ZIF-8 provided a strong therapeutic efficacy and possessed a persistent protective effect in SH-SY5Y cells. In comparison with other reports concerning about treatment of oxidative damage diseases based on ZIF-8 nanomaterials, our study exhibits satisfactory antioxidant duration and therapeutic effects (Table 1) (He, 2020; Zhang, 2022; Liu, 2021b). We believe that this study has important implications for the application of ZIF-8-based antioxidant nanomaterials in neurodegenerative diseases, as well as other diseases associated with oxidative damage.

**TABLE 1:** Study on the diagnosis and treatment of oxidization-related diseases based on ZIF-8 nanomaterials.

Nanomaterials	Particle sizes	Antioxidants	Antioxidant activity	Treatment of disease	References
CeO_2_@ZIF-8	About 100 nm	CeO_2_	SOD, CAT	ischemic stroke	He et al. (2020)
SOD@ZIF-8	About 150 nm	SOD	SOD	Noise-induced hearing loss	Zhang et al. (2022)
ZIF-8@PB-QCT	About 100 nm	PB-QCT	SOD, CAT	Parkinson’s disease	Liu et al. (2021b)

## Conclusion

In summary, SOD was successfully immobilized in the ZIF-8 through biomimetic mineralization method. The composite SOD@ZIF-8 showed satisfactory enzymatic activity and excellent stability against temperature, pH and ion environment. Moreover, compared with free SOD, SOD@ZIF-8 composites were capable of realizing efficient cellular uptake to scavenge intracellular ROS induced by MPP^+^ in neuroblasts, benefiting the cell survival state and cell activity significantly. Our study suggested that SOD@ZIF-8 composites could be employed as a potential approach for the treatment of nerve damage and other oxidative stress-stimulated neurological disorders.

## Methods

### Construction of SOD@ZIF-8 through biomimetic mineralization

The SOD@ZIF-8 composites through biomimetic mineralization was constructed according to previous reports (Fan et al., 2021; Zhu et al., 2022). Briefly, 2 ml of 2‐methylimidazole (HMeIM) solution (15.68 mg/ml) was mixed with 2 ml of Zn(OAc)_2_ solution (8.76 mg/ml), 200 μL of methanol and 1 ml of SOD solution (1 mg/ml). The compound was incubated at room temperature for 24 h, and then solids were collected by centrifugation and washed until no absorbance at 280 nm could be detected in the supernatant. Subsequently, solids were lyophilized to obtain SOD@ZIF-8 composites. The SOD loading in ZIF-8 was determine by BCA assay kit (Beyotime, Nanjing, China). Synthesis of FITC−SOD@ZIF-8.

20 mg SOD and 10 mg FITC were dissolved in 50 ml of PBS (50 mM, pH 8.0) and the mixture was stirred at room temperature for 24 h in the dark. The mixture was dialyzed against distilled water using Amicon^®^ Ultra-15 ultrafiltration tube (Millipore, MA) until no absorbance at 488 nm could be detected in the supernatant and then lyophilized to obtain FITC−SOD. Compound FITC−SOD@ZIF-8 was constructed as described in the above section.

### SOD enzymatic activity detection

SOD enzymatic activity was determined by nitro blue tetrazolium (NBT) assay and pyrogallol autoxidation assay. For NBT assay, solution containing 75 μM NBT, 13 mM methionine, 20 μM riboflavin, and free SOD or SOD@ZIF-8 were mixed in PBS buffer (25 mM, pH 7.4), respectively. The mixtures were detected at the absorbance at 560 nm using UV-2700 spectrophotometer (Shimadzu, Kyoto, Japan). For pyrogallol autoxidation assay, 240 μL enzyme solutions (1 mg/ml) was added to 750 μL Tris-HCL (pH 8.2) and incubated for 20 min 10 μL pyrogallol solution (50 mM) was then mixed into the enzyme solutions and the absorbance at 325 nm was measured for 1 min by UV-2700 spectrometer (Shimadzu, Kyoto, Japan). Characterization of SOD@ZIF-8 Composites.

After depositing samples evenly onto a rigorously cleaved silicon wafer surface, scanning electron microscopy (SEM) images and elemental analysis of SOD@ZIF-8 were obtained on a Hitachi FE-SEM S-4800 instrument with an acceleration voltage of 3 kV. Transmission electron microscopy (TEM) was carried on a JEM-2100F filed emission electron microscope with an acceleration voltage of 200 kV.

### Cell viability assay

SH-SY5Y cells were seeded into 96-well plates at a density of 7,000 cells/well overnight. Different formulations were added into the cells for 6 h and 2 mM MPP^+^ was subsequently added for 24 h. 20 μL of MTT solution (5 mg/ml) was added into each well and incubated at 37°C for another 4 h. Finally, dimethyl sulfoxide (DMSO) was used to dissolve the formed formazan crystal and the absorbance at 492 nm was measured by a GF-M3000 microplate reader (CAIHONG, Shandong, China).

### ROS detection of SH-SY5Y cells

ROS levels in SHSY-5Y cells were determined according to Reactive Oxygen Species Assay Kit (Beyotime, Nanjing, China). SH-SY5Y cells were seeded into 6-well plates at a density of 2.0×10^5^ cells per well and pre-treated with different formulations for 6 h. Cells were then exposed to 2 mM MPP^+^ for 2 h at 37°C and stained with 10 μM DCFH-DA for 20 min at 37°C. ROS expression was detected by flow cytometry (Accuri C6, Beckman, NJ) and analyzed using Beckman Coulter software.

### Statistical analysis

GraphPad Prism 8.0.2 was utilized for all statistical analyses. The outcomes were compared via Student’s *t*-tests. *p < 0.01, **p < 0.005, ***p < 0.001.

## Data Availability

The original contributions presented in the study are included in the article/supplementary material, further inquiries can be directed to the corresponding authors.
